# Novel aspects of the phosphorylation and structure of pathological tau: implications for tauopathy biomarkers

**DOI:** 10.1002/2211-5463.13667

**Published:** 2023-07-08

**Authors:** Taeko Kimura, Haruaki Sato, Maria Kano, Lisa Tatsumi, Taisuke Tomita

**Affiliations:** ^1^ Laboratory of Neuropathology and Neuroscience, Graduate School of Pharmaceutical Sciences The University of Tokyo Japan

**Keywords:** Alzheimer's disease, blood biomarker, cryo‐EM, phosphorylation, tau PET, tauopathy

## Abstract

The deposition of highly phosphorylated and aggregated tau is a characteristic of tauopathies, including Alzheimer's disease. It has long been known that different isoforms of tau are aggregated in different cell types and brain regions in each tauopathy. Recent advances in analytical techniques revealed the details of the biochemical and structural biological differences of tau specific to each tauopathy. In this review, we explain recent advances in the analysis of post‐translational modifications of tau, particularly phosphorylation, brought about by the development of mass‐spectrometry and Phos‐tag technology. We then discuss the structure of tau filaments in each tauopathy revealed by the advent of cryo‐EM. Finally, we describe the progress in biofluid and imaging biomarkers for tauopathy. This review summarizes current efforts to elucidate the characteristics of pathological tau and the landscape of the use of tau as a biomarker to diagnose and determine the pathological stage of tauopathy.

## Graphical abstract

This review discusses the deposition of aggregated tau protein, which is a characteristic of tauopathies including Alzheimer's disease. Recent advances in analytical techniques have revealed the biochemical and structural differences of tau deposits in each tauopathy. Here, we focus on the analysis of post‐translational modifications, tau filament structure, and the development of biofluid and imaging biomarkers for tauopathy.

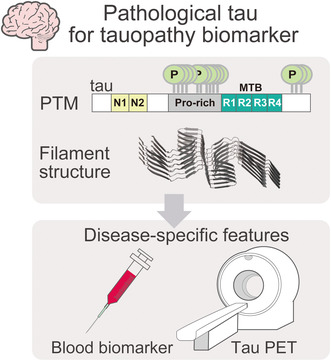

**Abbreviations**
AD: Alzheimer's disease
AGD: argyrophilic grain disease
Aβ: amyloid‐β
CBD: corticobasal degeneration
Cdk5: cyclin‐dependent kinase 5
CSF: cerebrospinal fluid
CTE: chronic traumatic encephalopathy
FBD: familial British dementia
FDD: familial Danish dementia
FTDP‐17: frontotemporal dementia with parkinsonism‐17
GGT: globular glial tauopathy
GSK3β: glycogen synthase kinase‐3β
MAP: microtubule‐associated protein
MT: microtubule
MTB: microtubule‐binding
NFTs: neurofibrillary tangles
PART: primary age‐related tauopathy
PHFs: paired helical filaments
PiD: Pick's disease
PP2A: protein phosphatase 2A
PSP: progressive supranuclear palsy
PTMs: post‐translational modifications
SFs: straight filaments
SSPE: subacute sclerotic panencephalitis

Tau is a brain‐specific microtubule (MT)‐associated protein (MAP) first described by Weingarten *et al*. [1]. Tau is mainly localized in neuronal axons where it stabilizes axonal microtubules and then regulates axon outgrowth and axonal transport. These physiological functions of tau are regulated by phosphorylation with different protein kinases [2, 3]. Tau is a heat‐stable molecule consisting of 441 amino acids in the case of the longest human tau, consisting of three regions, an N‐terminal projection domain and an MT‐binding (MTB) domain, and C‐terminal tail regions. Alternative mRNA splicing of the N‐terminal insertions of exons 2 and 3 and the second MTB domain of exon 10 produces six isoforms, all of which are expressed in the adult human brain. In particular, alternative splicing of exon 10 results in the generation of tau isoforms with either a three‐repeat (3R) or four‐repeat (4R) MTB domain [4]. 4R tau has stronger MT binding and assembly ability than 3R tau. Tau has attracted much attention since hyperphosphorylated tau (p‐tau) was identified as a major component of neurofibrillary tangles (NFTs), a pathological hallmark of Alzheimer's disease (AD) [5, 6, 7, 8]. While an amyloid‐β (Aβ) deposition is linked to the pathological pathway of AD, there is also evidence that the frequency and distribution of NFTs are highly correlated with neuronal loss and progression of clinical symptoms [9, 10]. In addition, the pathological role of tau has been intensively studied since the discovery of causative tau mutations in frontotemporal dementia with parkinsonism‐17 (FTDP‐17) in 1998. More than 40 mutations in the *MAPT* gene are now known [11, 12]. There are many other neurodegenerative diseases in which deposits of hyperphosphorylated tau are found. These tau‐involved diseases are collectively referred to as tauopathies and include AD, primary age‐related tauopathy (PART), FTDP‐17, corticobasal degeneration (CBD), progressive supranuclear palsy (PSP), Pick's disease (PiD), argyrophilic grain disease (AGD), globular glial tauopathy (GGT) and chronic traumatic encephalopathy (CTE) [13, 14]. In addition to AD, tau deposits are found in the disease characterized by extracellular amyloid deposition; familial British dementia (FBD), familial Danish dementia (FDD), and prion disease. Interestingly, the aggregated tau in the brains of patients with tauopathies is highly phosphorylated in common, but there are known differences in the isoforms and cell types in which tau is deposited. Therefore, elucidating the differences in tau aggregates at the molecular level in each of these diseases will lead to understanding the pathogenic mechanism of each tauopathy.

## Phosphorylation of tau deposited in the patients' brains

Biochemical analyses of the tau filaments obtained from the patients' brains revealed several post‐translational modifications (PTMs), such as phosphorylation, ubiquitination, and acetylation. However, to date, phosphorylation is the most common PTM in deposited tau filaments and is utilized as pathological diagnostics of tauopathy.

### Overview of tau phosphorylation

Tau phosphorylation is regulated by a balance between kinases [such as cyclin‐dependent kinase 5 (Cdk5), glycogen synthase kinase‐3β (GSK3β), and protein kinase A (PKA)] and phosphatases [such as protein phosphatase 2A (PP2A)] [15]. In postmortem brains of patients with tauopathies, this balance is disturbed, and tau is hyperphosphorylated. More than 50 tau phosphorylation sites have been reported in the brains of AD patients [3]. It has been suggested that phosphorylation of tau causes a conformational change in the protein and promotes the shedding of tau from microtubules by removing the positive charge of the MTB region [16]. Although it is controversial whether phosphorylation is a cause or a consequence of tau aggregation, the study of tau phosphorylation is key to understanding pathology. Since tau was identified as a component of NFT, many phosphorylation‐specific antibodies against tau, such as AT8 (pS202/pT205) and PHF1 (pS396/pS404), have been used as markers of pathological tau. However, it has not been possible to estimate protein phosphorylation patterns or total phosphorylation levels although relative phosphorylation levels can be analyzed using p‐tau antibodies. Here, we summarize the phosphorylation of tau in healthy and diseased brains, focusing on analysis by phosphate‐binding tag (Phos‐Tag) SDS/PAGE method and quantitative analysis by mass spectrometry (MS).

### Phos‐tag SDS/PAGE analysis of tau phosphorylation in cultured cells

**Fig. 1:**
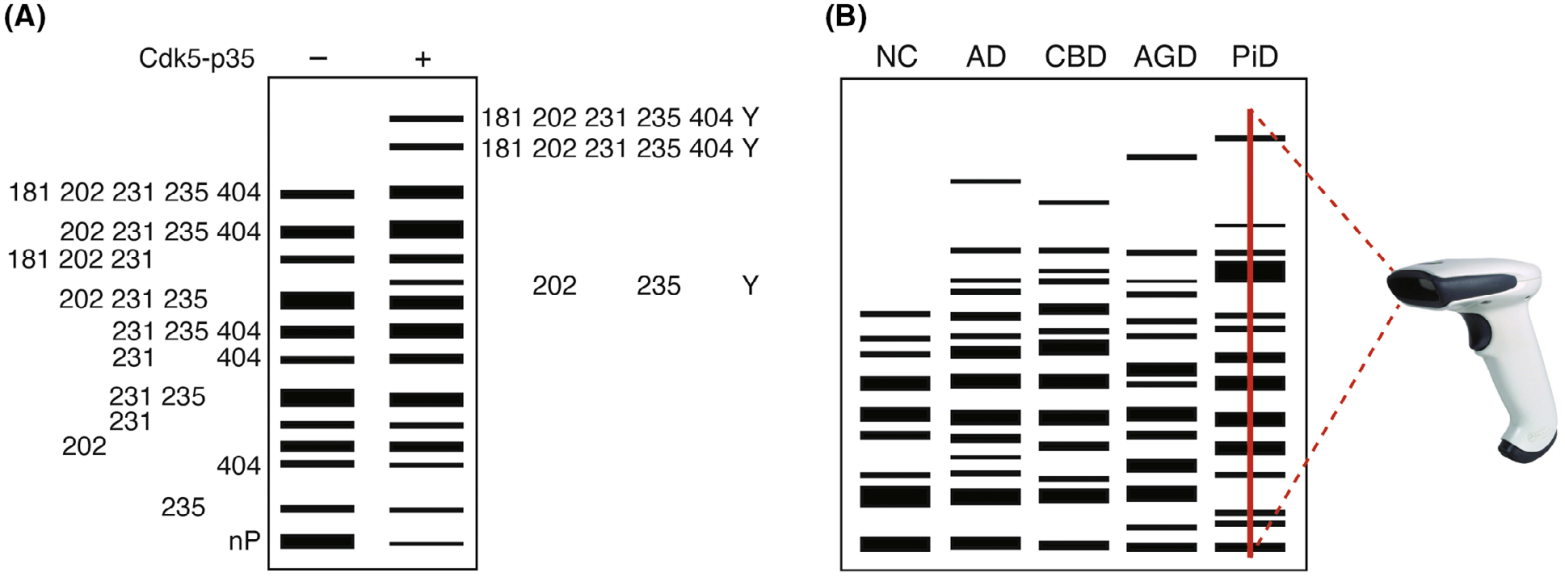
Phos‐tag SDS/PAGE analysis of tau phosphorylation. (A) Separation of phosphorylated tau by Cdk5‐p35 in cultured cells using Phos‐tag SDS/PAGE analysis. Numbers indicate the phosphorylated amino acid residues. nP means nonphosphorylated tau. Y represents unidentified phosphorylated site(s). (B) ‘Phosphorylation barcode’ of tau deposited in the different tauopathy brains. We propose that a simple diagnosis of tauopathy will be possible by reading banding patterns created by Phos‐tag SDS/PAGE with a barcode reader.

Phos‐tag SDS/PAGE allows distinguishing phosphorylated from nonphosphorylated tau by verifying the level of phosphorylation by the position of bands on the gel. Using this technique, the combination of phosphorylation sites (isotypes) and their level can be quantitatively estimated [17]. *In vitro* experiments revealed that normal tau has 12 different phosphorylation isotypes, with the major sites being T181, S202, T231, S235, and S404 (Fig. 1A) [18]. It was also found that phosphorylated tau in the combination of pS202, pT231, and pS235 was present at a high rate of 20.4% and that T231 was phosphorylated in approximately 50% of total tau molecules.

In the soluble fraction of the normal human brain, the three‐repeat (3R) and four‐repeat (4R) isoforms of tau without N‐terminal inserts have low molecular weights and can be isolated from the phosphorylated bands, allowing analysis of their presence. Of the respective isoforms, 17.9% and 14.1% were unphosphorylated, suggesting that unphosphorylated tau is present to some extent. Detection of tau isolated by the Phos‐tag method with the pS404 antibody showed that 57% of the total tau was phosphorylated. Quantitative analysis experiments using MS with stable isotope labeled tau standards in the normal human brain were also performed and showed that more than 50% phosphorylation occurred for S404, similar to the Phos‐Tag SDS/PAGE data [19]. Phos‐Tag SDS/PAGE was then used to compare phosphorylation in AD, CBD, AGD, PSP, PiD, and other tauopathies. In the human brain, all six isoforms are expressed, and the banding pattern is complex. Therefore, a comparison was made using the banding pattern of Phos‐tag SDS/PAGE. The results showed different banding patterns among tauopathies, suggesting the presence of disease‐specific phosphorylation [18, 20, 21]. Immunoblotting with phosphorylated antibodies revealed differential site‐specific phosphorylation in the temporal lobes of patients with different tauopathies. Increased pS202, pT231, and pS235 were observed in the AD brains, increased pS202 in the brains with PiD, and increased pS396 in the brains with AGD. In the future, the ‘tau phosphorylation barcode’, a phosphorylation‐dependent banding pattern in Phos‐tag SDS/PAGE shown by tau, is expected to be useful for understanding the pathogenesis and diagnosis of tauopathies (Fig. 1B).

### Quantitative tau phosphorylation analysis among tauopathies using MS

**Fig. 2:**
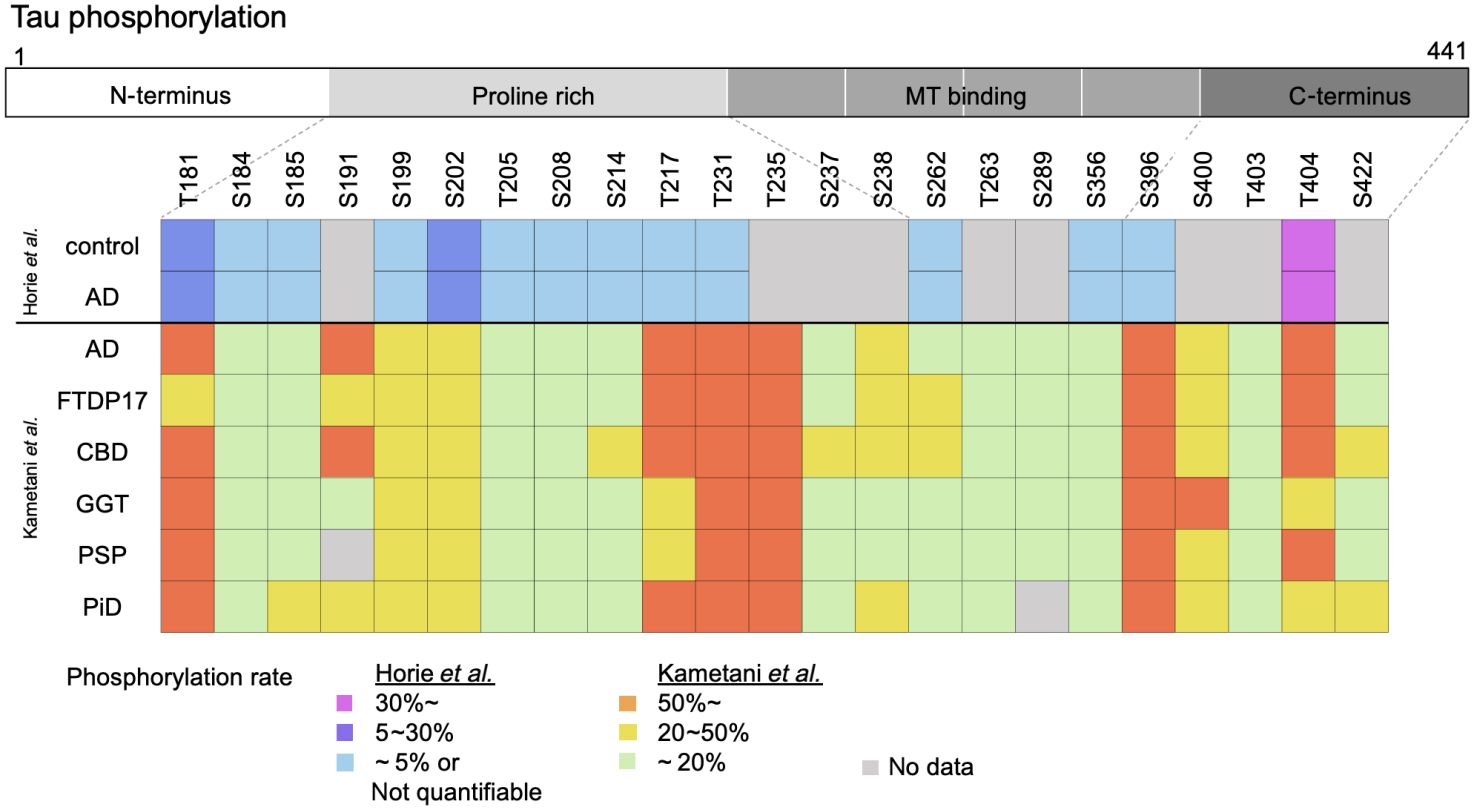
Phosphorylation patterns of tau deposited in the tauopathy brains. The figure shows the phosphorylation sites of tau identified in each disease and the frequency (Horie *et al*. and Kametani *et al*. [19, 23]).

A large study compared the frequency of tau phosphorylation in 49 AD patients with 42 healthy controls and found AD‐specific phosphorylation at T217 and S262 [22]. In addition, phosphorylation can be compared quantitatively by determining the percentage of phosphorylated peptides among the total number of peptides containing each amino acid residue by MS. Phosphorylation sites that are more enriched in AD were found to be T111, S113, T153, T181, S199, S202, T205, T217, T231, S262, and S396 in the insoluble fraction compared with healthy control. And the effect of Aβ on tau PTM has also been studied, and T111, T153, and T217 were found as phosphorylation sites that are promoted with Aβ42 accumulation in the AD patient brains (Fig. 2) [19]. In a paper by Kametani *et al*. [23], phosphorylation analysis of insoluble tau revealed characteristic phosphorylation in each tauopathy. In AD, pS191 was as high as > 50%, whereas pS356 was barely detectable. In PiD, pS262 was absent. These could be related to the respective tau filament structures. In addition, T181, T217, T231, S235, S396, and S404 were highly phosphorylated at > 30% in all tauopathy patients (Fig. 2).

There are several challenges to tau phosphorylation studies in the human brain. One of them is postmortem time. In mice, dephosphorylation has been reported to occur at 1‐min postmortem [24]. In human brains, tau is dephosphorylated during postmortem delay, even though aggregated tau is relatively resistant to dephosphorylation. Some studies have used biopsy samples from human brains, and a decrease in body temperature due to anesthesia during surgery also alters tau phosphorylation [25, 26]. In other words, the exact phosphorylation state of tau in the human brain is not yet known. MS also analyzes proteins in fragments, making it difficult to identify tau isoforms. In addition, the relationship between tau PTMs and filament formation should be further discussed.

## Structural analysis of tau filaments from the brain of tauopathy patients

**Fig. 3:**
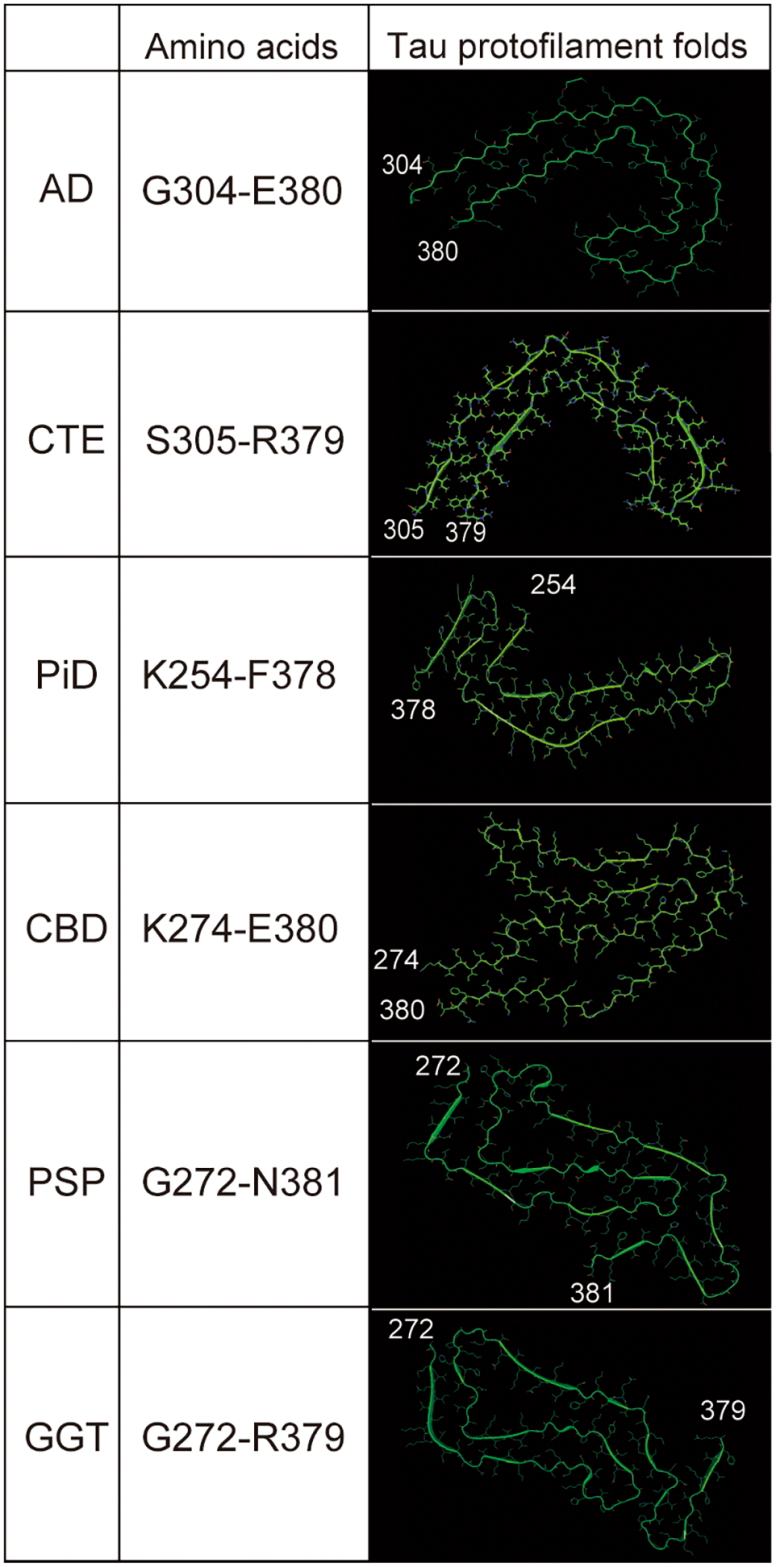
Structures of the core of tau filaments revealed by cryo‐EM analysis. Ribbon diagram of the cores of tau filaments. Numbers indicate amino acid residues in the 2N4R tau sequence. Each tau protofilament structure is visualized by pymol, Schrodinger, New York, NY,USA). Following EMDB datasets are used: AD, EMD‐21207; CTE, EMD‐14024; PiD, EMD‐0077; CBD, EMD‐10514; PSP, EMD‐13218; GGT, EMD‐13219. AD: PHFs and SFs share a C‐shaped core composed of residues G304‐E380. CTE: The cores of type I and II filaments are common structures like AD fold composed of S305‐R379 consistent with SSPE. They are differ from AD by a hydrophobic cavity around E338‐I354. PiD: NPFs and WPFs have a common J‐shaped core consisting of K254‐F378 of only 3R tau. CBD: Type I and II share the largest four‐layered core like AGD core among tauopathies consisting of K274‐E380. It contains a cavity surrounded by K290, K294 and K370. PSP: PSP cores have a three‐layered folded structure consisting of residues G272‐N381. Each repeat is systematically bent. Three additional density gaps are between N279 and G323, K294 and D314 and, K317, K321, and K340. GGT: GGT cores have three‐layered structure like PSP fold composed of G272‐R379.

Tau filaments have disease‐specific morphologies, and their formation proceeds through the generation of disease‐specific stable protofilament cores and the subsequent assembly of multiple cores. It is proposed that stable protofilament core structures determine tau filament structure, propagation, and neuropathic potential [27]. In recent years, remarkable advances in cryo‐electron microscopy (cryo‐EM) have established the structure‐based classification of tauopathy (Fig. 3) [28, 29].

### AD fold

The formation of NFTs in AD begins in the locus coeruleus to the transentorhinal cortex and progressively affects the hippocampus, temporal cortex, and polymodal association areas [30]. There are two types of tau filaments, paired helical filaments (PHFs) and straight filaments (SFs), which are commonly found in AD [31, 32]. They are composed of all six isoforms of 3R and 4R tau. Paired helical filaments are formed by two twisted, symmetrically coupled protofilaments, whereas SFs are asymmetrically coupled linear filaments. They share a common core structure with the same C‐shaped core composed of residues G304‐E380 (Fig. 3, AD), including repeat 3 (R3) and repeat 4 (R4) of the tau MTB domains, called AD fold [33, 34]. The difference between PHFs and SFs structures lies in the contact surfaces of the two protofilaments: in PHFs, the paired helical structures are formed at the P332‐Q336 interface; in SFs, the two protofilaments stack asymmetrically at the K317‐S324 or P312‐K321 interface. Furthermore, regardless of clinical or pathological progression, PHFs and SFs are remarkably identical in sporadic and inherited AD patients [35].

Further cryo‐EM studies revealed the possible mechanism of the formation of AD fold tau filament. Primary age‐related tauopathy is a neuropathological designation with features distinct from AD, and develops independently of Aβ plaques [34]. Notably, not only the biochemical character but also the structure of PART filaments was identical to that of AD. Furthermore, tau filaments with AD fold were identified in the brains of FBD, FDD, and prion disease [28, 36]. These diseases are associated with the extracellular deposition of amyloids composed of distinct peptides: British amyloid, Danish amyloid, and prion proteins. Thus, these similarities suggest that the AD fold of tau filaments was induced by age and extracellular amyloids.

### CTE fold

In CTE, tau filaments are found in neurons and astrocytes around small blood vessels in the depths of cortical sulci [37, 38]. Tau filaments are composed of all six isoforms, 3R and 4R, as in AD, and cryo‐EM analysis revealed that CTE filaments have two types of helical filaments, 90% of which are type I: a helical filament [39]. The remaining 10% of filaments (type II) have a paired helical structure with a pronounced helical twist. They are analogous to the core of the AD fold. The cores are composed of K274‐R379 of R3 and S305‐R379 of R4. They differ from AD fold by a hydrophobic cavity around residues E338‐I354 (Fig. 3, CTE). The presence of a cofactor in this cavity has been suggested, which could be some ions that leak from blood vessels due to injury damage. Recently, it has been suggested that sodium ions could be the most plausible cofactors, as discussed in detail in the next section [28, 40].

Subacute sclerosing panencephalitis (SSPE) is a neurological disease following exposure to the measles virus and is described as a type of tauopathy that develops at a young age. Filamentous inclusions consist of 3R and 4R tau that accumulate in neurons and glial cells in the superficial cortex of the limbic system. Recent cryo‐EM analysis revealed that the core structure of SSPE has a structure similar to that of CTE fold, with common protofilaments of the two types of filaments, type I accounting for more than 90% of the filaments observed and type II for less than 10%, similar to those observed in CTE (Fig. 3, CTE) [41]. As CTE and SSPE are chronic diseases, the inflammatory response might be involved in the formation of CTE fold.

### PiD fold

Spherical tau filaments, called pick bodies, are seen in neurons in degenerative areas of the cortex in the frontotemporal lobes of PiD patients. Filaments composed of a single 3R isoform [29, 42]. Pick body‐derived tau filaments are of two types, narrow and wide pick filaments (NPFs and WPFs). Narrow pick filaments (93%) are linear with twists, and WPFs (7%) are formed by two NPF protofilaments joined at their distal tips by van der Waals interactions. They share a common J‐shaped protofilament core structure consisting of K254‐F378 of 3R tau, including R1, R3, and R4. PiD is unique among tauopathies as only 3R tau aggregates and the structure is quite different from other tauopathies (Fig. 3, PiD). Thus, a 3R‐specific disease‐dependent mechanism would underlie the formation process of PiD fold [42].

### CBD fold

The filaments are composed of only 4R tau observed not only in perivascular neurons, but astrocytes as astrocytic plaque in the cortex, brainstem, and basal ganglia [43]. They are observed in cell types similar to PSP. It accumulates in neurons at degenerative sites in the cortex. There are two types of helically twisted CBD tau filaments: narrow filaments (type I) and broad filaments (type II), paired inversely parallel to type I [44]. Type II is common in CBD patients. Both have in common that the core protofilaments are four‐layered folded, consisting of K274‐E380, the last residue of R1 R2, R3, the entire R4, and the 12 amino acids after R4. The CBD core is the largest among other tauopathies. The CBD core also contains a nonprotein‐dense cavity. It is surrounded by side chains from K290 and K294 in R2 and K370 in R4 and has a positively charged hydrophilic space (Fig. 3, CBD). In the brains of CBD patients, filaments are particularly observed in perivascular astrocytes, suggesting that cofactors may enter the brain from the periphery [28].

Argyrophilic grain disease is late‐onset dementia morphologically characterized by the presence of abundant spindle‐shaped argyrophilic grains in neuronal processes and coiled bodies in oligodendrocytes. Biochemical studies revealed that AGD is a 4R tauopathy similar to PSP and CBD [29]. Intriguingly, the structure of tau filaments of AGD adopts a four‐layered ordered structure that resembles CBD fold [28]. Also, other 4R tauopathies, such as aging‐related tau astrogliopathy and FTDP‐17 linked to *MAPT* intron‐10 mutations +3 and +16, develop the tau filaments with CBD fold.

### PSP fold

Tau aggregation and neuronal degeneration in PSP begin in neurons of the pallido‐nigro‐luysian axis and spread to the brainstem, basal ganglia, and cortex. Tau filaments are composed of 4R and accumulate in neurons, oligodendrocytes, and astrocytes as tufted astrocytes [43, 45, 46]. However, the structure of the tau filament in PSP was distinct from that of the CBD fold. PSP cores have a three‐layered folded structure consisting of residues G272‐N381 of 4R tau (Fig. 3, PSP) [28]. Each repeat is bent with a common sequence PGGG at the C‐terminus, with R3 as the central layer and R2 and R4 storing R3. Three additional density gaps are observed. One is between N279 and G323, surrounded by hydrophobic side chains and possibly containing nonpolar molecules. The second is between K294 and D314 and is most likely a solvent molecule. The remaining one is located at the R3 and R4 interface, between positively charged K317, K321, and K340, and may correspond to an anion molecule. Notably, GGT, another 4R tauopathy, also develops tau filaments with PSP fold‐like three‐layered structure(Fig. 3, GGT), suggesting the similarities in the pathogenic process of these diseases [28].

### Reconstitution of tau filaments

Tau is not an aggregation‐prone protein. The N‐terminal domain, including the positively charged proline‐rich region, and the C‐terminal domain inhibit the spontaneous assembly of full‐length recombinant tau. Therefore, recombinant full‐length tau assembly requires polyanionic assembly accelerators *in vitro*, such as heparin and dextran sulfate [47, 48]. However, they have different structures from those in human patients, making it difficult to elucidate the molecular mechanisms underlying human tauopathies [49, 50].

Based on cryo‐EM studies above, it has become possible to reproduce patient filaments using ‘cores’. Several amino acid sequences that induce AD‐like filaments have been reported, including AD core sequences and portions of the proximal amino acids. Appropriate shaking and buffering conditions can produce tau filaments with PHF structures in the absence of cofactors [40, 51, 52]. The antibodies that recognize the recombinant AD tau core filaments recognize tau filaments in AD patients. This suggests that they may also share common structures. In addition, the cavities in CTE type II filaments may correspond to the sodium and chloride ions in the buffer. These ion levels are much lower than 200 mm in neurons, but damage‐specific increases in concentration have been reported [40]. Nevertheless, these structural biology findings by cryo‐EM facilitate the understanding of the pathogenic process of each tauopathy.

## Biomarkers for tauopathy

**Fig. 4:**
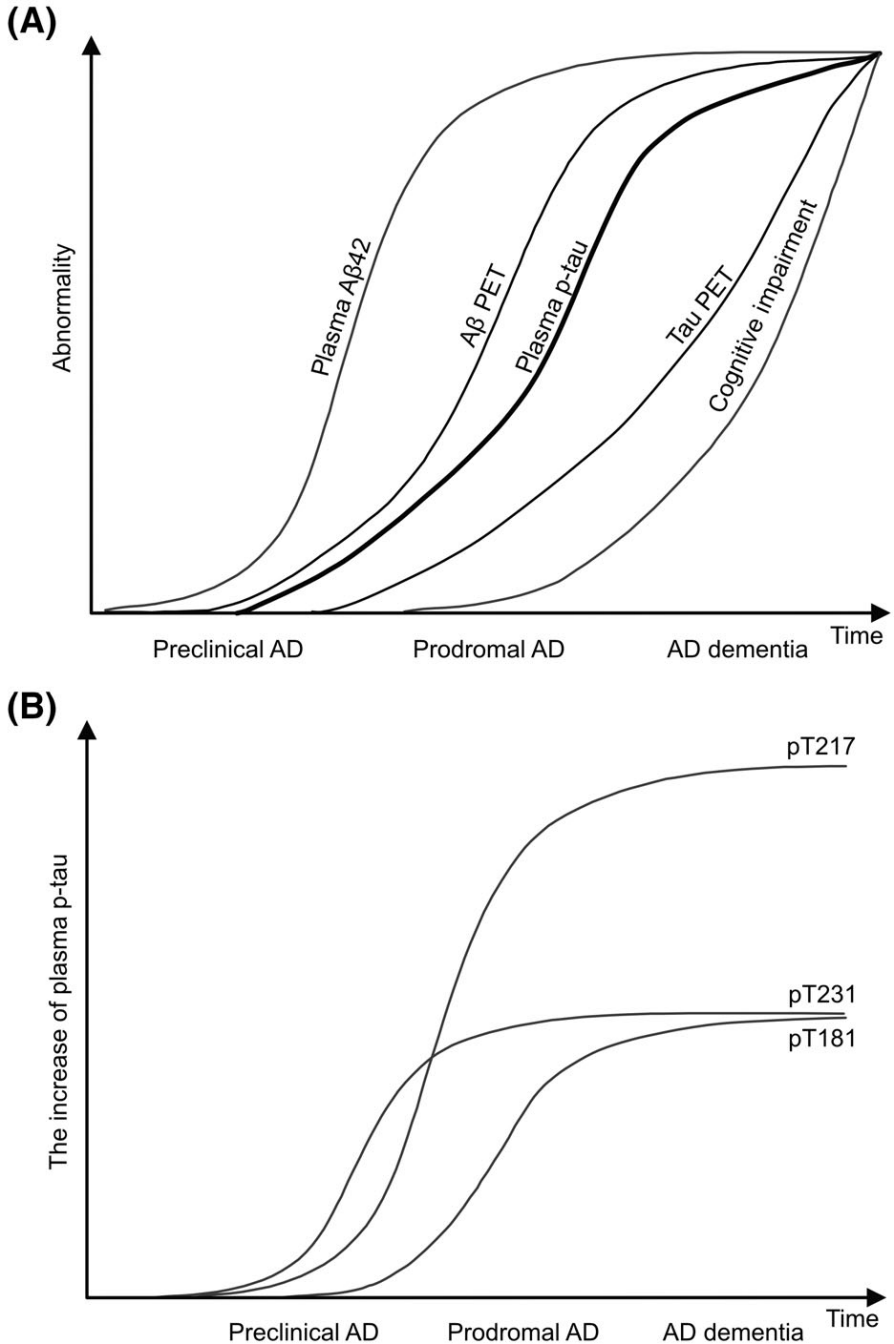
Trajectory and biomarker changes of AD patients. (A) Schematic representation of pathological changes in the brain and plasma of AD patients. (B) Schematic representation of changes of phosphorylated tau in plasma of AD patients.

Tauopathies have overlapping clinical symptoms and pathologic features [53]. Therefore, it is difficult to make an accurate diagnosis based on clinical symptoms or cognitive function tests. In addition, appropriate intervention methods differ according to the pathological progression of tauopathy. For example, the pathological process of AD follows many processes, from Aβ accumulation, which begins decades before the onset of the disease, to tau aggregation, neuronal death, and subsequent cognitive decline [54]. Therefore, there is an urgent need to discover effective biomarkers that can discriminate among diseases with similar symptoms and identify the specific pathological stage. Biochemical and structural differences in the aggregated tau would provide an opportunity to establish biomarkers that are specific to each of the tauopathies. Among the tauopathies, AD is the most extensively studied disease (Fig. 4) [55, 56]. To date, amyloid PET and Aβ levels in cerebrospinal fluid (CSF), as well as plasma, are known biomarkers of Aβ accumulation in the brain [57, 58]. For tau deposition, tau PET and tau levels in CSF, as well as plasma, have been extensively tested. Notably, phosphorylated and truncated forms of tau in biofluids have attracted attention because their increase occurs before tau aggregation and after Aβ accumulation, describing the more detailed pathological stage. In this section, we will discuss tau‐based AD biomarkers and compare them to those in another tauopathy.

### Biofluid biomarker

The levels of p‐tau in CSF have been examined as a biomarker for AD and primary tauopathies including carriers with *MAPT* mutation [56, 59, 60], but CSF collection is invasive. Therefore, a method accurate and sensitive enough to detect p‐tau in plasma, which is easier to collect but small in amount, has been searched for a decade. The modified MS technology has revealed a variety of p‐tau species that correlate with AD pathology. And it has also shown that those p‐tau species are N‐terminal to midfragments and are different from the tau fragments that accumulate in the AD brain [61]. In addition, improved ELISA assays, such as Meso Scale Discovery (MSD) as well as single molecule array (Simoa) assays, allow the measurement of p‐tau at concentrations as low as fg·mL^−1^ and have been used to validate the availability of these p‐tau species [61, 62, 63, 64].

pT181, pT217, and pT231 have been mainly investigated as biomarkers of AD [61, 64, 65, 66, 67, 68, 69, 70, 71, 72]. All these p‐tau species show high accuracy in discriminating between Aβ‐positive and Aβ‐negative patients even in the preclinical phase, high specificity for AD, and prognostic potential. Among them, pT181 is the most established species. Anti‐Aβ treatment, such as Aducanumab, in AD patients reduced CSF pT181 [66], suggesting a causal relationship between increased plasma pT181 and Aβ accumulation since CSF pT181 levels correlate with plasma levels. pT217 is more sensitive than pT181 in specifically detecting Aβ‐positive patients, and its increase in plasma occurs earlier than pT181, indicating a higher sensitivity to Aβ accumulation. In addition, the amount of pT231 in plasma increases earlier than pT217 or pT181, although no firm conclusions can be drawn about its accuracy. Importantly, the dynamic range of pT217 is generally greater than that of pT231 and pT181. Therefore, the measurement of multiple p‐tau species together with Aβ in plasma is important, and a composite biomarker would be required for accurate diagnosis of AD.

Other biomarkers such as pT205 [73], pS235 [74], brain‐derived tau‐specific isoforms [75], N‐terminal tau [76, 77, 78, 79, 80], and midfragments of tau in CSF [81] are also expected to serve as AD biomarkers. Especially, MTBR domain fragments in CSF have recently been shown to potentially reflect tau accumulation in the AD brain [82] and may be elevated specifically in 4R tauopathy [59]. In contrast, total tau in plasma does not correlate with that in CSF, thus making it unsuitable as a biomarker [73]. The mechanism by which biomarkers change remains to be elucidated. Previous studies have shown that the amount of p‐tau in CSF or plasma correlates with Aβ PET and tau PET, but Aβ PET shows more correlation [83, 84, 85, 86]. We recently revealed that sAPPβ, which is a proteolytic fragment of amyloid precursor protein in the Aβ production pathway, positively regulates tau secretion in cultured cells [87]. As sAPPβ production is upregulated around Aβ plaques [88], this mechanism might be involved in the biological relationship between Aβ deposition and tau in biofluids.

### Imaging biomarkers

Tau PET is a promising biomarker of tau aggregation because it can visualize tau aggregates in the brain while the patient is alive. The major first‐generation tau PETs were developed in 2013, and one of them, [^18^F]AV‐1451 (Flortaucipir), was approved by the FDA as a biomarker of tau aggregation [89, 90, 91]. Subsequently, several second‐generation tau PET tracers have been developed, such as [^18^F]RO‐948 and [^18^F]MK‐6420. In general, the reactivity of these tracers is limited to patients with AD and CTE, in which 3R and 4R mixed tau aggregates are deposited. It is consistent with the structural similarity of tau filaments of AD and CTE, as revealed by cryo‐EM studies. In addition, [^18^F]PI‐2620 and [^18^F]PM‐PPB3/APN‐1607 (Florzolotau) can bind to different tau aggregates independent of tau isoforms, such as those derived from primary tauopathies with only 4R tau aggregates. As the brain region with tau deposits differs from disease to disease, these tracers have the potential to discriminate tauopathies based on imaging data [92, 93, 94, 95, 96, 97]. However, as recently shown in AD, the signal pattern shows heterogeneity even in the same disease using the same tracer [98]. Therefore, further imaging studies together with other tracers would be needed to achieve accurate diagnostics. Furthermore, recent structural analyses of tau filaments might also lead to the development of novel tracers targeting disease‐specific tau aggregates. Based on the structure of tau filaments resolved by cryo‐EM, the binding sites of [^18^F]PM‐PBB3 ligand to tau filaments were identified by cryo‐EM analysis [34]. Also, simulation analyses suggested that the binding sites of the tracers to tau filaments from each type of tauopathy showed a unique pattern due to the difference in structure [99, 100, 101, 102, 103, 104]. However, experimental structural analysis for these filaments and tracers is needed to confirm these putative binding sites. Nevertheless, modifying the tracer to bind to the specific structure of tau aggregates in each tauopathy is another solution for identifying the disease.

## Concluding remarks and future prospectives

Recent progress in biochemical and structural analysis has shed light on the pathological properties of tau deposits in the brain. Plasma p‐tau species and tau PET are promising biomarkers for tauopathy patients. Nevertheless, it is becoming apparent that these diseases are chronic, progressive conditions that require early diagnosis and intervention. Understanding the disease continuum and the relevance of biochemical and structural changes that contribute to abnormal tau metabolism, aggregation, deposition, and ultimately neurodegeneration is essential. Furthermore, the reasons why tau proteins with the same primary sequence undergo distinct folding, phosphorylation, and other modifications that are specifically observed in each tauopathy remain unclear. Therefore, it is critical to analyze the contribution of nonprotein components observed in cryo‐EM structural analyses of tau filaments. Innovative approaches will be crucial in providing insight into the diagnosis and treatment of tauopathies, which account for a significant portion of neurodegenerative diseases.

## Conflict of interest

The authors declare no conflict of interest.

## Author contributions

TK, HS, LT, and MK wrote the draft and revised it. TT was involved in discussing, drafting, and editing the manuscript. All authors approved the submitted version.
